# Supplementary material for: Sir proteins impede, but do not prevent, access to silent chromatin in living Saccharomyces cerevisiae
Source: Sci Rep. 2026 Apr 28;16:14730. doi: 10.1038/s41598-026-44518-0 (PMC13161209; doi:10.1038/s41598-026-44518-0)
Supplement: Supplementary file 1 — Supplementary Material 1 [file 41598_2026_44518_MOESM1_ESM.pdf]

Supplementary Information

Sir proteins impede, but do not prevent, access to silent chromatin in living *Saccharomyces cerevisiae*

Kenneth Y. Wu, Zhuwei Xu, Hemant K. Prajapati, Peter R. Eriksson and David J. Clark

Division of Developmental Biology, *Eunice Kennedy-Shriver* National Institute of Child Health and Human Development, National Institutes of Health, Bethesda MD 20892, USA

Supplementary Figures S1-S9.

Supplementary Table S1.

Supplementary References.

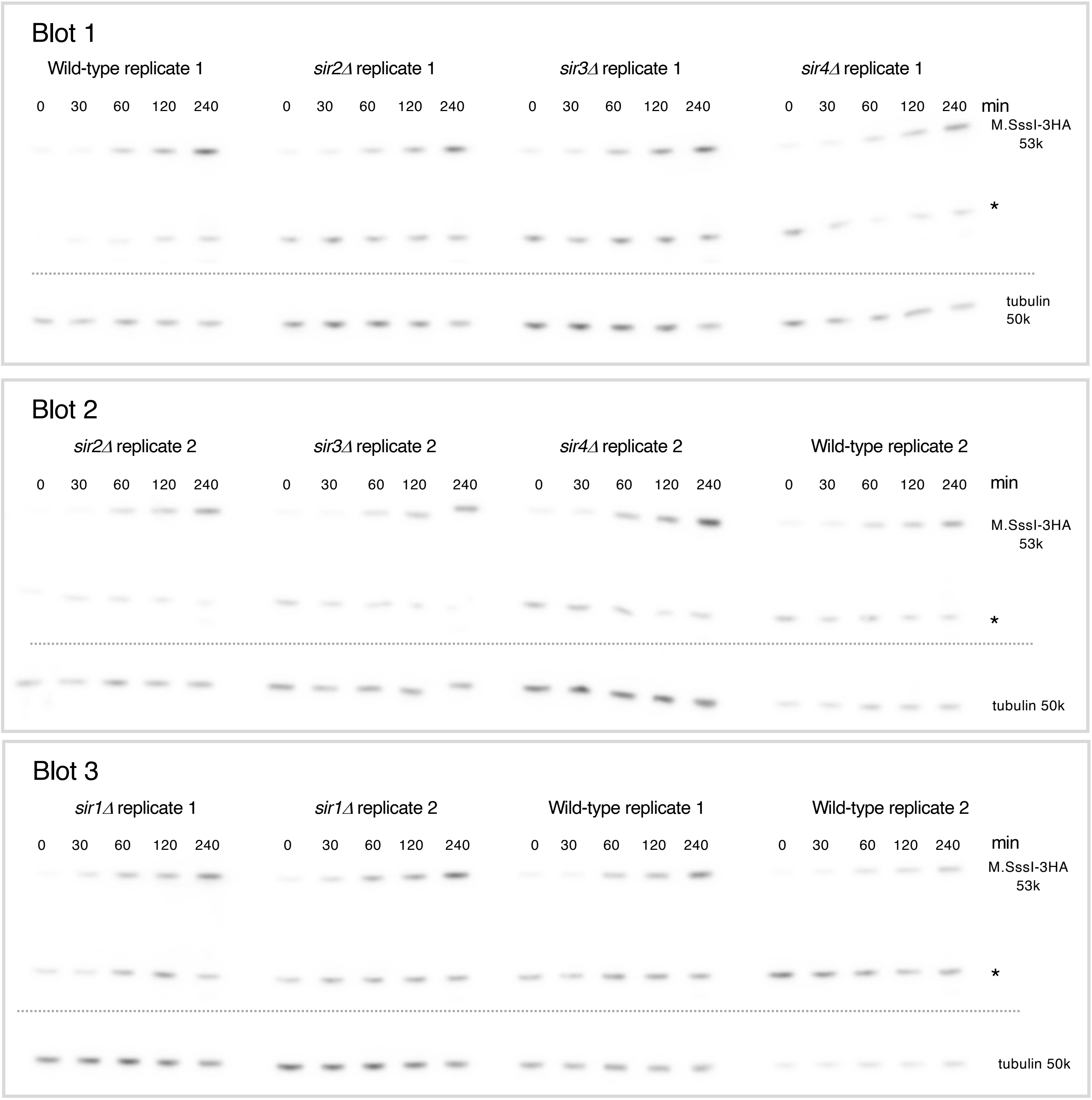

Supplementary Figure S1. SM induction of M.SssI expression in replicate wild-type, *sir1Δ*, *sir2Δ*, *sir3Δ* and *sir4Δ* experiments. Western blots for HA-tagged M.SssI and for tubulin across time course samples for each strain. Each blot contains four time courses, probed first for M.SssI-3HA (upper rows) and then for tubulin (lower rows). * non-specific band.

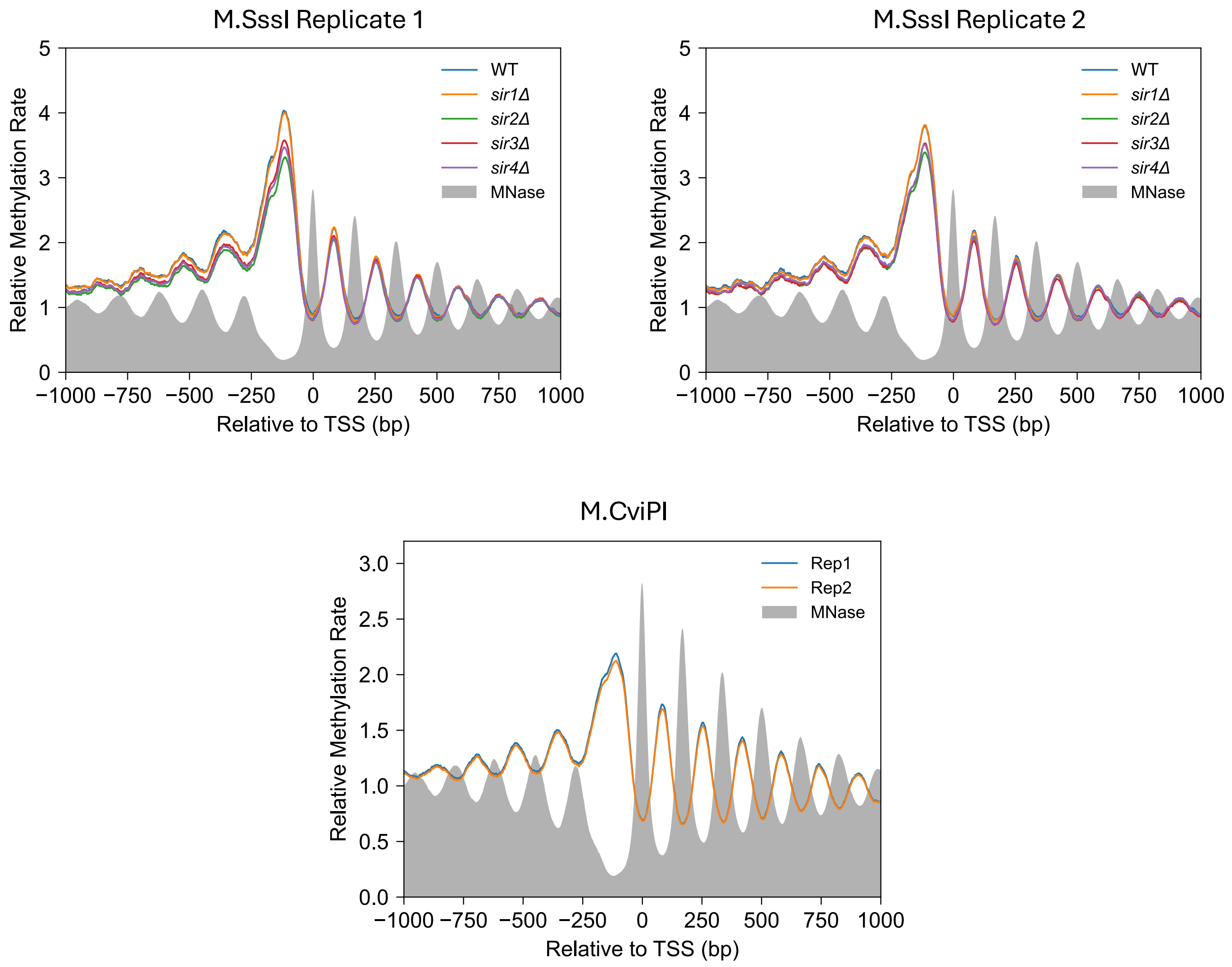

Supplementary Figure S2. Global nucleosomal phasing at genes is unaffected by the absence of Sir1, Sir2, Sir3 or Sir4 in living cells. Nucleosomal phasing can be detected from aggregate M.SssI methylation rates across all genes aligned to the +1 nucleosome for wild-type, *sir1Δ*, *sir2Δ*, *sir3Δ* and *sir4Δ* strains. Data for M.CviPI methylation rates in wild-type cells are also shown. MNase-seq data for the same regions in a wild-type strain are shown in grey.

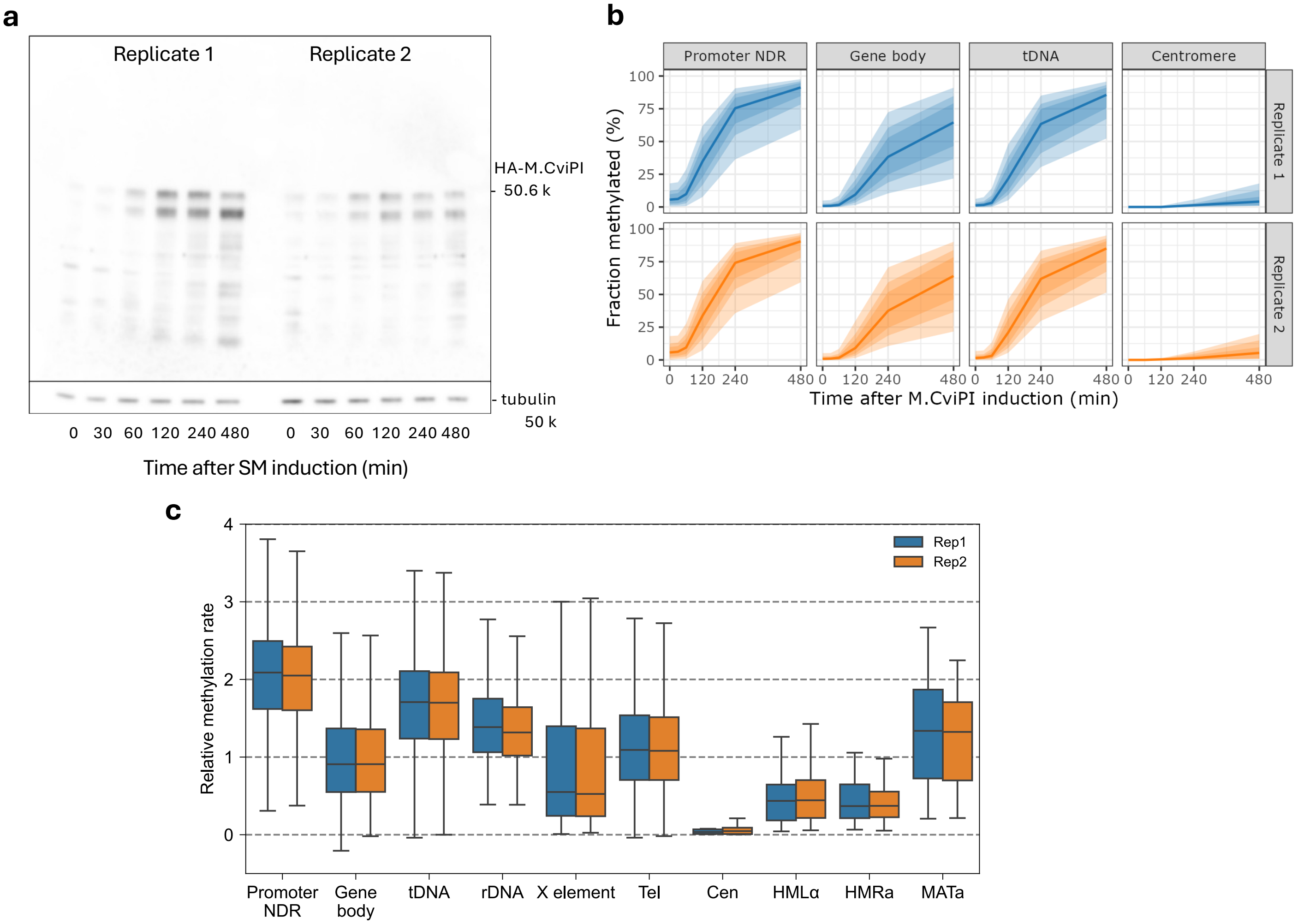

d

Rep	Prom. NDR	Gene body	tDNA	rDNA	XE	TEL	CEN	*HMLα*	*MATa*	*HMRa*
1	2.1	0.9	1.7	1.4	0.6	1.1	0.0	0.4	1.3	0.4
2	2.1	0.9	1.7	1.3	0.5	1.1	0.1	0.4	1.3	0.4

Supplementary Figure S3. M.CviPI methylation assays mirror M.SssI methylation assays. (**a**) Western blot for HA-tagged M.CviPI and tubulin. Two biological replicate experiments were compared in the same blot. (**b**) M.CviPI methylation time courses showing the median GC site methylation (solid line) for promoter NDRs, gene bodies, tDNA and centromeres. Shading: lightest to darkest: 5-95%, 15-85% and 25-75% of all GC sites in the feature. (**c**) Box plots showing the distributions of methylation rate constants for all individual GC sites in each genomic feature. Boxes contain 25 to 75% of the data, the line is the median and the whiskers represent 1.5 times the interquartile range to the farthest data points. (**d**) Table of median methylation rate constants of GC sites for each genomic feature (XE: X-elements).

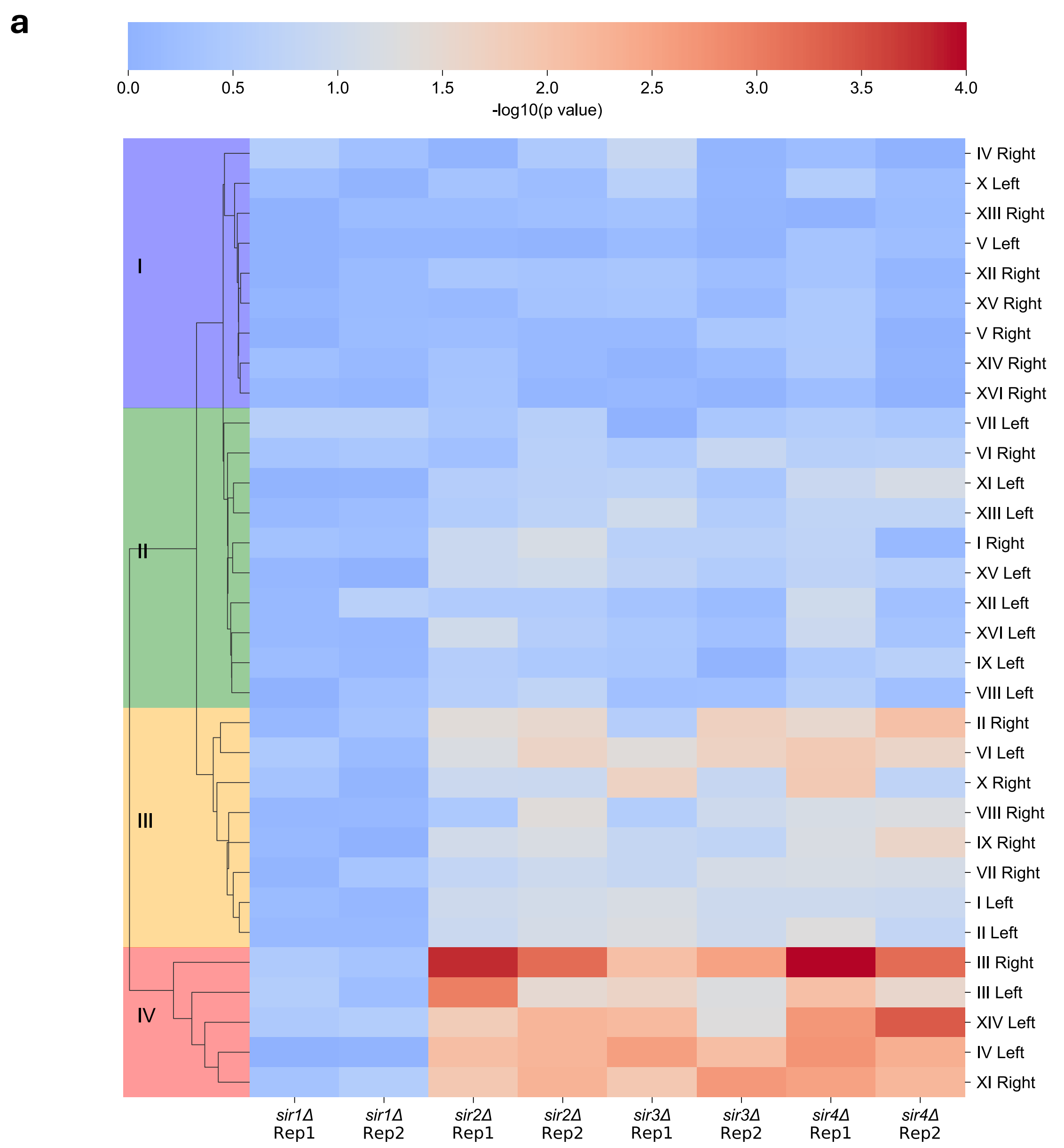

See following pages for b, c and d.

Supplementary Figure S4. X-element classification and methylation rate differences. (**a**) Heatmap showing -log10(p values) from Mann-Whitney *U* tests comparing X-element methylation rates of individual CpG sites for pooled wild-type (replicates 1 and 2) and separate *sir* mutant replicates. Each column represents the comparison between pooled wild-type and one biological replicate of the indicated *sir* mutant. Each row represents one X-element. The colormap is centered at p = 0.05 (-log10(0.05) = 1.3), with blue indicating p > 0.05 (insignificant) and red indicating p < 0.05 (significant). We derived four clusters of X- elements (I-IV). (**b** – *next page)* Box plot analysis of methylation rate constants for each X- element (organized by the clusters in **a**). (**c** – *following page*) Box plots showing the distances of X-elements from the chromosome end in each cluster. X-elements in cluster I are all > 5 kb from the chromosome end. (**d** - *following page*) X-element sequence identity matrix (Clustal Omega). No strong correlation between methylation rate cluster and sequence identity.

b

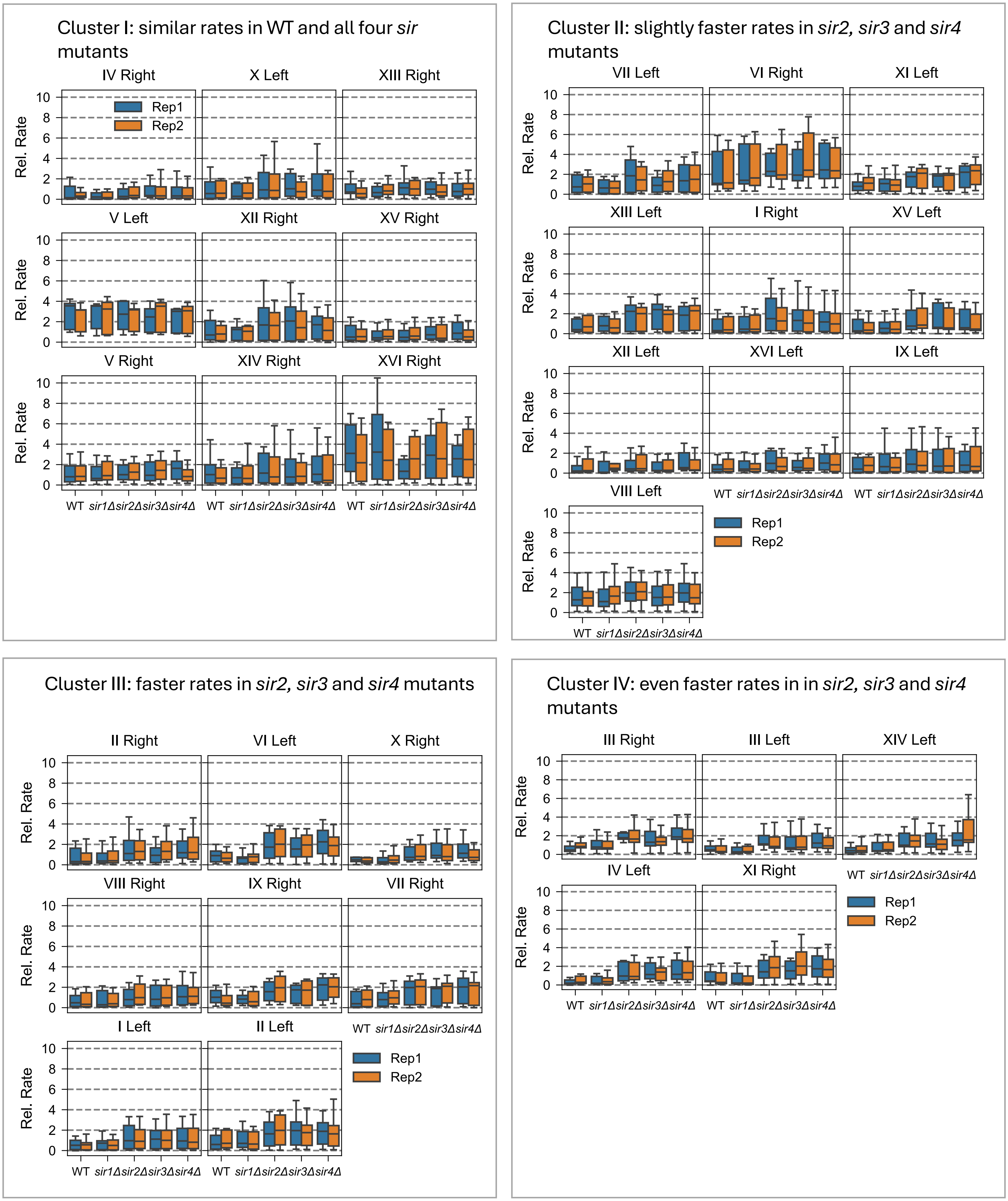

Supplementary Figure S4, *continued*.

c

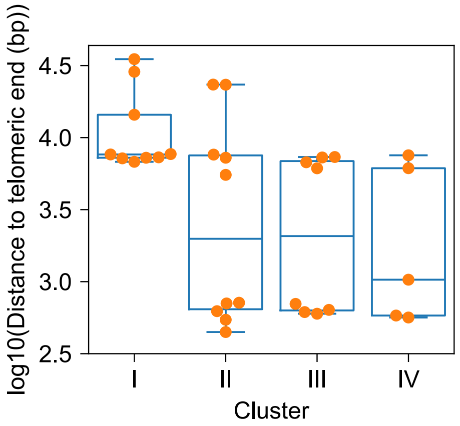

d

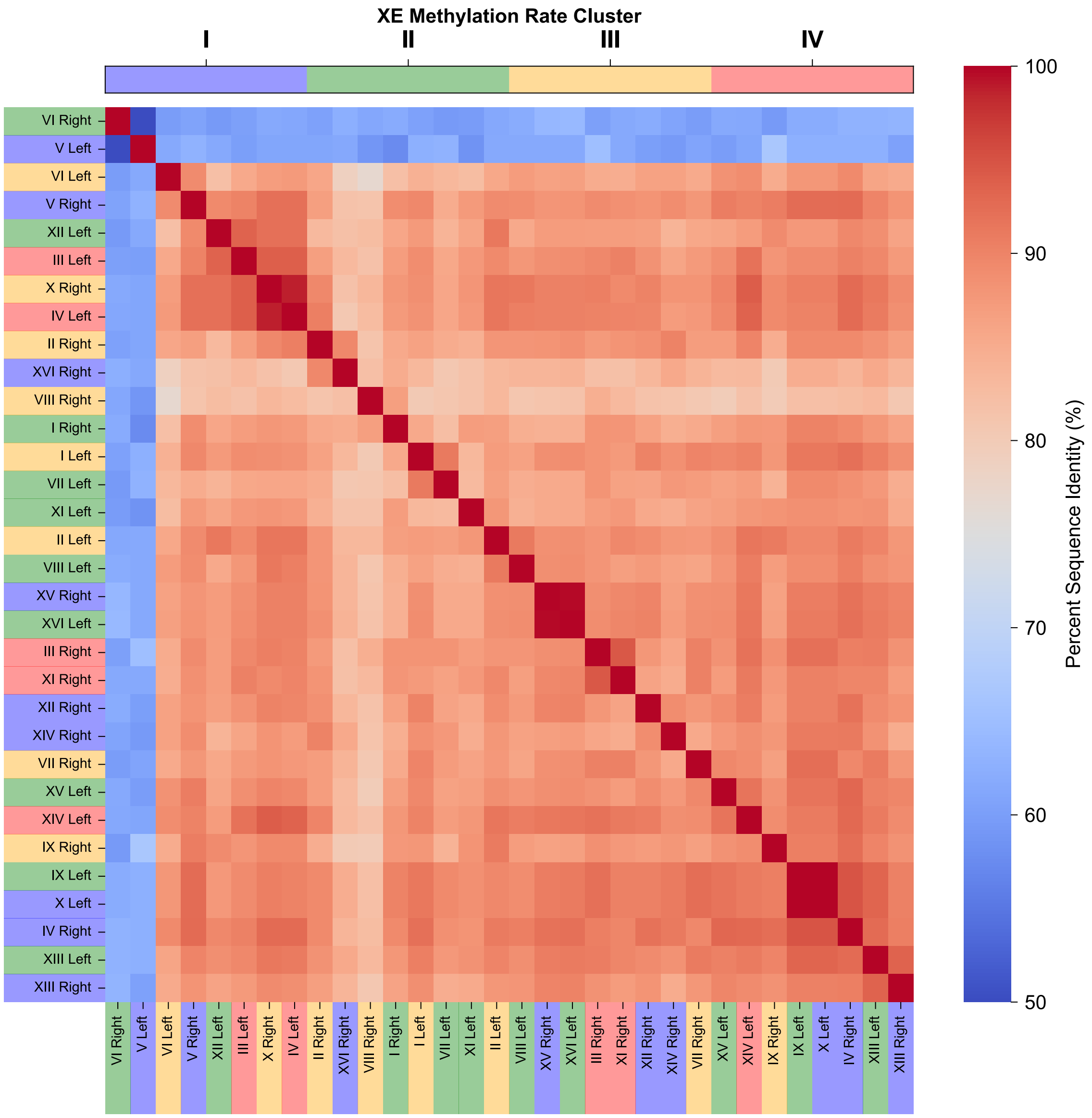

Supplementary Figure S4, *continued*.

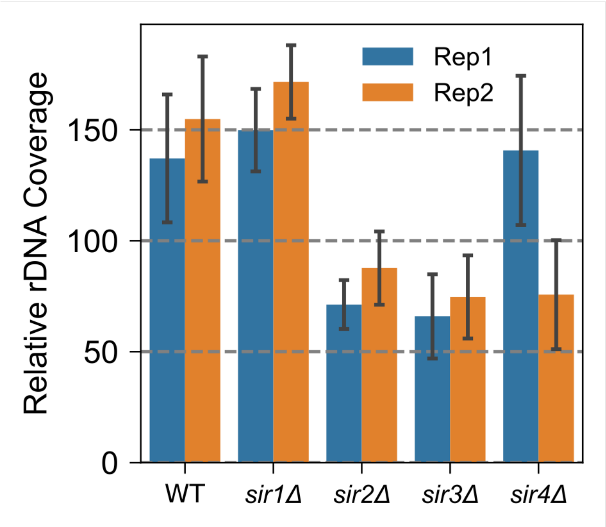

Supplementary Figure S5. rDNA copy number in *sirΔ* strains. Copy numbers were estimated by dividing the rDNA sequence coverage by the genome average (excluding chromosome XII). The average relative rDNA coverage for all five time points is shown for each replicate, with error bars indicating the standard deviation.

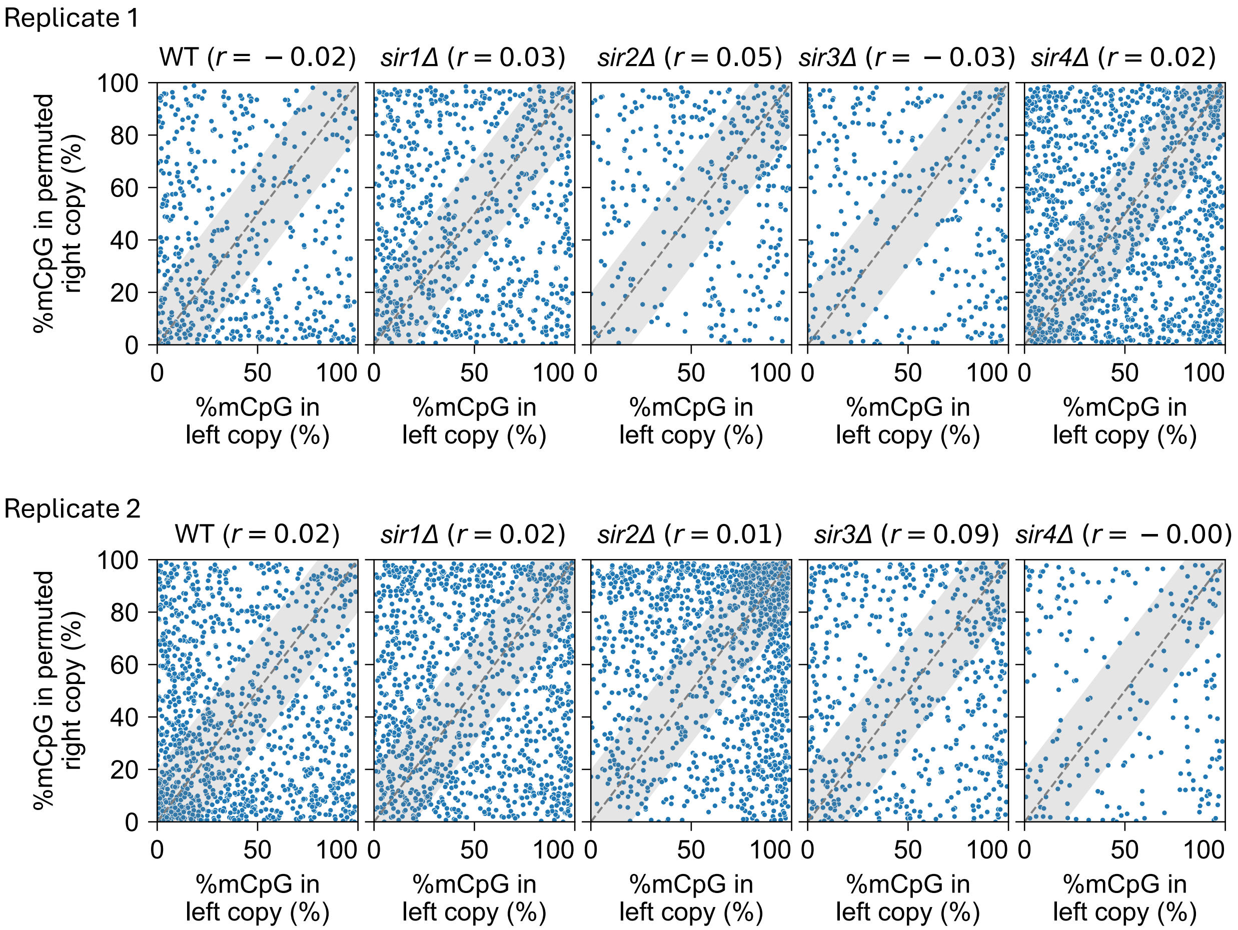

Supplementary Figure S6. Permutation controls for the correlation between M.SssI methylated fractions for adjacent *RDN37* genes in the same nanopore read (see Fig. 4a). Methylated fractions of the left *RDN37* copy were randomly shuffled while the methylated fractions of the right copy were unaltered. The data were then re-plotted to assess the significance of the observed left-right correlation. The grey area indicates data within 20% of the diagonal.

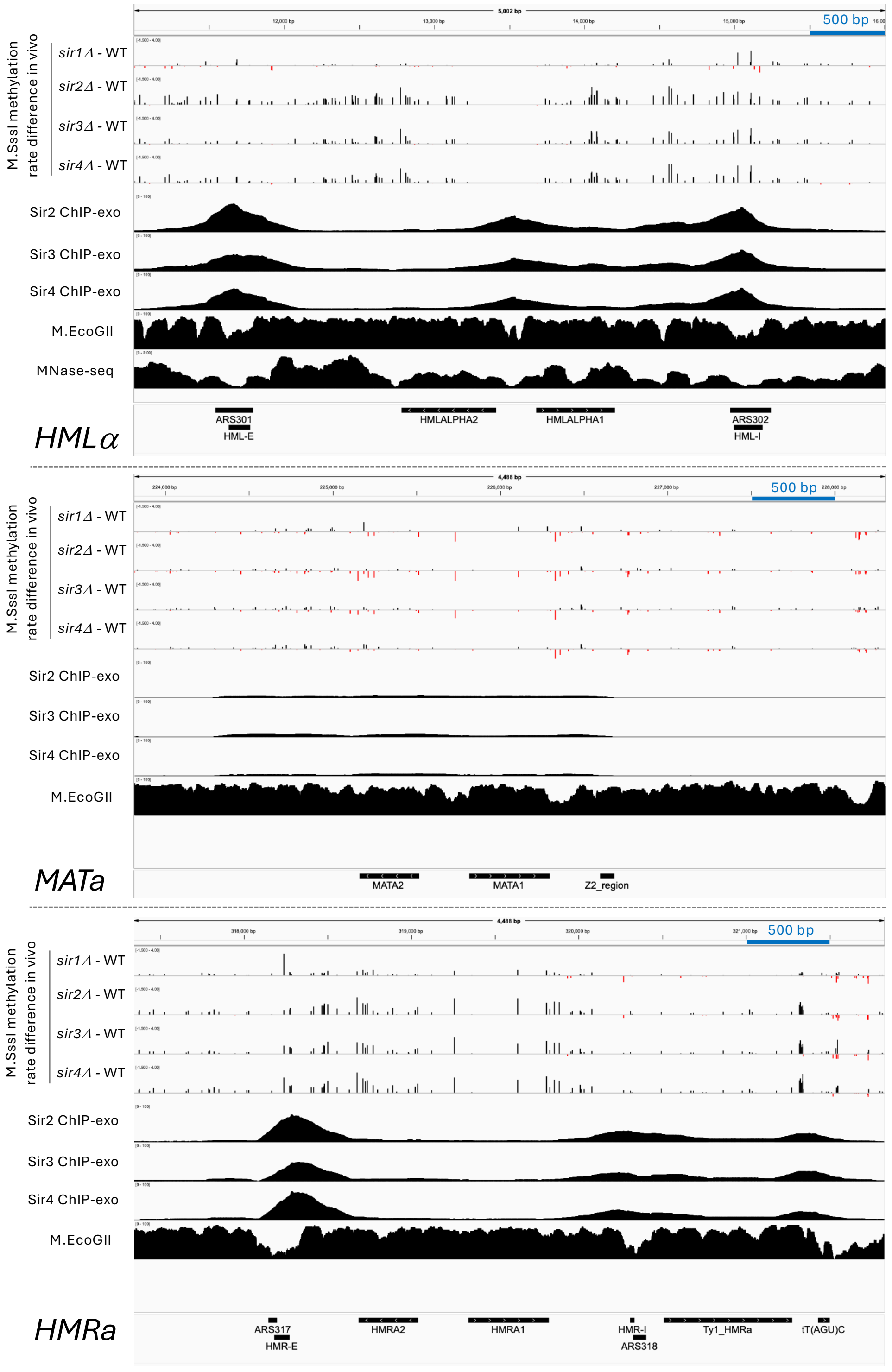

Supplementary Figure S7. Differential methylation rates at CG sites in *HMLα*, *MATa* and *HMRa* relative to Sir protein location. IGV tracks. For each CG site, the M.SssI methylation rate in wild-type (WT) cells was subtracted from that in *sir1Δ*, *sir2Δ*, *sir3Δ* or *sir4Δ* cells. Black columns above the line: faster in the mutant; red columns below the line: slower in the mutant. M.EcoGII data for wild-type nuclei are from Dennis et al. (2024) [1]. MNase-seq data for wild-type nuclei are from Ocampo et al. (2016) [2]. ChIP-exo data for the Sir proteins are from Rossi et al. (2021) [3].

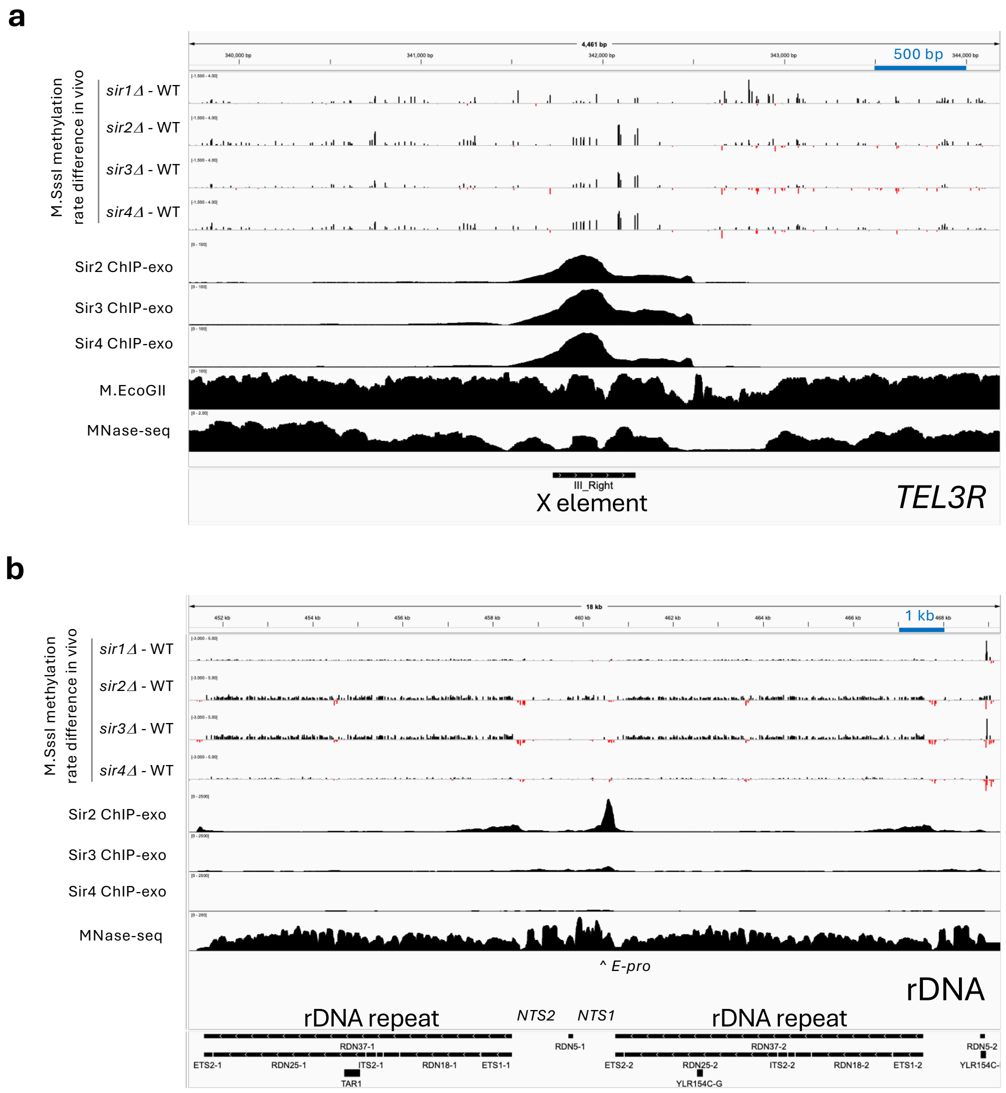

Supplementary Figure S8. Differential methylation rates at CG sites relative to Sir protein location. (**a**) *TEL3R* (the telomere at the righthand end of chromosome III) including the X element. (**b**) Two rDNA repeats (chromosome XII).
IGV tracks. For each CG site, the M.SssI methylation rate in wild-type (WT) cells was subtracted from that in *sir1Δ*, *sir2Δ*, *sir3Δ* or *sir4Δ* cells. Black columns above the line: faster in the mutant; red columns below the line: slower in the mutant. M.EcoGII data for wild-type nuclei are from Dennis et al. (2024) [1]. MNase-seq data for wild-type nuclei are from Ocampo et al. (2016) [2]. ChIP-exo data for the Sir proteins are from Rossi et al. (2021) [3].

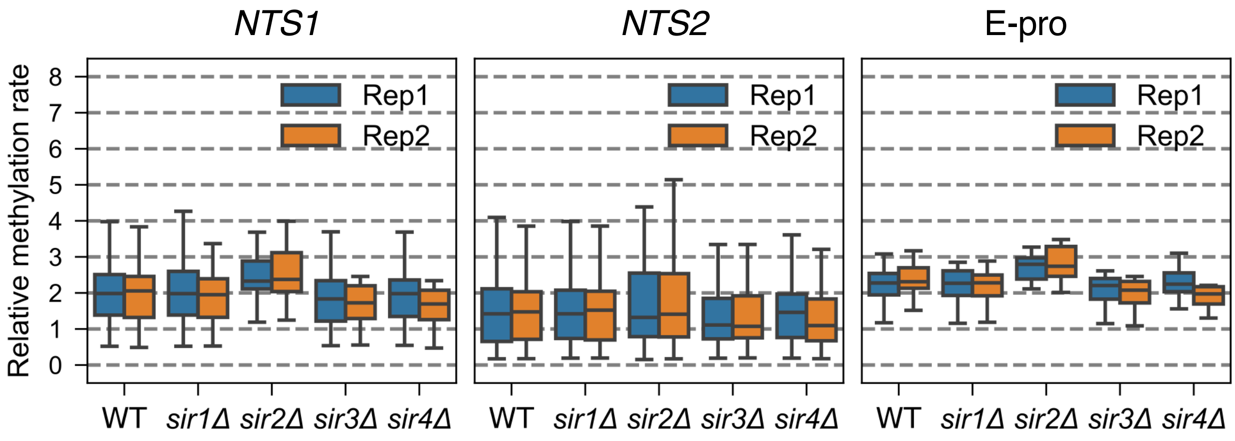

Supplementary Figure S9. Box plots showing the distributions of methylation rate constants of individual CG sites located within the *NTS1, NTS2* regions and the E-pro promoter in the rDNA repeats. Boxes contain 25 to 75% of the data, the line is the median and the whiskers represent 1.5 times the interquartile range to the farthest data points.

Supplementary Table S1. Yeast strains used in this study.
All strains are derived from a W303 *RAD5+* strain (Kim et al., 2006) [4].

Strain	Genotype
**YDC111**	*MATa ade2-1 can1-100 leu2-3,112 trp1-1 ura3-1*
**YHP827**	*MATa ade2-1 can1-100 leu2-3,112 trp1-1 ura3-1 ho::TIR1_MSssI-degron-3HA_KanMX (p906)*
**YPE840**	*MATa ade2-1 can1-100 leu2-3,112 trp1-1 ura3-1 ho::TIR1_MSssI-degron-3HA_KanMX (p906) sir2Δ::Hph (p923)*
**YPE841**	*MATa ade2-1 can1-100 leu2-3,112 trp1-1 ura3-1 ho::TIR1_MSssI-degron-3HA_KanMX (p906) sir3Δ::Hph (p924)*
**YPE842**	*MATa ade2-1 can1-100 leu2-3,112 trp1-1 ura3-1 ho::TIR1_MSssI-degron-3HA_KanMX (p906) sir4Δ::Hph (p925)*
**YHP853**	*MATa ade2-1 can1-100 leu2-3,112 trp1-1 ura3-1 ho::TIR1_NLS-degron-3HA-MCviPI_KanMX (p956)*
**YKW874**	*MATa ade2-1 can1-100 leu2-3,112 trp1-1 ura3-1 ho::TIR1_MSssI-degron-3HA_KanMX (p906) sir1Δ::NatNT2 (p994)*

Supplementary References

1. Dennis, A. F., Xu, Z. & Clark, D. J. Examining chromatin heterogeneity through PacBio long-read sequencing of M.EcoGII methylated genomes: an m6A detection efficiency and calling bias correcting pipeline. Nucleic Acids Res 52, e45, doi:10.1093/nar/gkae288 (2024).
2. Ocampo, J., Chereji, R. V., Eriksson, P. R. & Clark, D. J. The ISW1 and CHD1 ATP-dependent chromatin remodelers compete to set nucleosome spacing in vivo. Nucleic Acids Res 44, 4625-4635, doi:10.1093/nar/gkw068 (2016).
3. Rossi, M. J. et al. A high-resolution protein architecture of the budding yeast genome. Nature 592, 309-314, doi:10.1038/s41586-021-03314-8 (2021).
4. Kim, Y., McLaughlin, N., Lindstrom, K., Tsukiyama, T. & Clark, D. J. Activation of Saccharomyces cerevisiae HIS3 results in Gcn4p-dependent, SWI/SNF-dependent mobilization of nucleosomes over the entire gene. Mol Cell Biol 26, 8607-8622, doi:10.1128/mcb.00678-06 (2006).
